# The Association between Hepcidin and Iron Status in Children and Adolescents with Obesity

**DOI:** 10.1155/2021/9944035

**Published:** 2021-06-26

**Authors:** Ekkarit Panichsillaphakit, Orapa Suteerojntrakool, Chitsanu Pancharoen, Issarang Nuchprayoon, Sirinuch Chomtho

**Affiliations:** ^1^Division of Nutrition, Department of Pediatrics, King Chulalongkorn Memorial Hospital, Thai Red Cross Society, Bangkok 10330, Thailand; ^2^Division of Nutrition, Department of Pediatrics, Surin Hospital, Surin 32000, Thailand; ^3^Ambulatory Division, Department of Pediatrics, Faculty of Medicine, Chulalongkorn University, Bangkok 10330, Thailand; ^4^Pediatric Nutrition Research Unit, Division of Nutrition, Department of Pediatrics, Faculty of Medicine, Chulalongkorn University, Bangkok 10330, Thailand; ^5^Infectious Division, Department of Pediatrics, Faculty of Medicine, Chulalongkorn University, Bangkok 10330, Thailand; ^6^Palliative Care Unit, Department of Pediatrics, Faculty of Medicine, Chulalongkorn University, Bangkok 10330, Thailand

## Abstract

**Introduction:**

Iron deficiency (ID) is the most common nutritional deficiency found in pediatric practice. A higher prevalence of ID may be found in children with obesity. Obesity is a chronic low-grade inflammatory condition. It is postulated that inflammation increases hepcidin, a regulator of iron homeostasis. The aim of this study was to investigate the associations between iron status, hepcidin, and BMI-standard deviation score (BMI-SDS) in children with and without obesity.

**Methods:**

A cross-sectional study of Thai children with obesity (5 to 15 years old) versus age- and sex-matched, nonobese controls was conducted. A total of 63 children with obesity and 27 controls were enrolled. Complete blood count, serum iron, ferritin, transferrin saturation, and total iron binding capacity were analyzed. Serum hepcidin-25 was assayed using a hepcidin ELISA Kit (Human Hepc25).

**Results:**

There were 63 children with obesity, the median age (IQR) being 10 (9–13) years, and 27 controls. The median (IQR) BMI-SDS of the obese group was 2.3 (2.0–2.6) vs. −0.5 ((−1.3)−0.4) of the control group. ID was diagnosed in 27 children in the obese group (42.9%); 4 of the children with obesity and ID had anemia. Serum hepcidin-25 levels of the children with ID vs. without ID in the obese group were not significantly different (median (IQR) 25 (12.9–49.2) and 26.4 (12.6–43.6), respectively) but both of them were significantly higher than controls (19.7 (8.3–25.5) ng/ml, *p* = 0.04). BMI-SDS was positively correlated with hepcidin-25 (*r* = 0.28, *p* = 0.001).

**Conclusion:**

Prevalence of iron deficiency in Thai children with obesity and serum hepcidin-25 was higher than controls. Further study in a larger population, preferably with interventions such as weight loss program, is warranted to clarify this association.

## 1. Introduction

Obesity has an adverse impact on public health. The inverse relationship between iron status and adiposity was first reported in 1962 by Wenzel et al. [1]. Iron deficiency (ID) is associated with decreased exercise capacity, impaired cognitive function, developmental delays, and behavioural disturbances [2–4]. Children and adolescents with obesity may be at risk of ID because they tend to consume unbalanced meals, which are particularly rich in carbohydrate and fat but low in nutritional values [2, 5].

The 3^rd^ US National Health and Nutrition Examination survey found a higher prevalence of ID in children that were overweight [6]. A previous study reported a prevalence of ID in children with obesity (38.8%) was much higher than children of normal weight (4.4%) [2]. A study from Moayeri et al. [7] found that the prevalence of ID in children with obesity (body mass index, BMI ≥ 95^th^ percentile) was 6.9% and 5.3% in children that were overweight (BMI ≥ 85^th^ percentile), compared with 2.5% in children of normal weight. In Thailand, Yimyaem et al. demonstrated that 28% of the children with obesity (*n* = 50) had ID and 8% had iron deficiency anemia (IDA) [8].

Obesity is a chronic low-grade inflammatory condition that stimulates inflammatory cytokine release, such as tumor necrosis factor (TNF-*α*), interleukin-6 (IL-6), and C-reactive protein (CRP) [9–12]. These cytokines directly promote hepcidin expression [13]. Hepcidin is a 25-amino-acid peptide that is produced mainly by the liver, secreted into plasma, and excreted in urine. It is the main regulator of systemic iron homeostasis which restricts the intestinal iron absorption and iron release from macrophages [14–16]. Hepcidin also controls ferroportin (an iron transporter) expression in target cells [17, 18]. A previous study suggested that patients with chronic diseases had higher hepcidin concentrations, causing decreased iron absorption and increased iron sequestration in the reticuloendothelial system which resulted in anemia [19]. Studies from Western countries reported higher serum hepcidin-25 in children with obesity and some of them revealed an association of serum hepcidin-25 with iron profiles [15, 20, 21]. However, there were no reports in the Southeast Asian children which may uncover diverse associations. This study aimed to assess iron status and serum hepcidin-25 in Thai children and adolescents with obesity and investigate their relationships with body mass index-standard deviation score (BMI-SDS).

## 2. Materials and Methods

### 2.1. Participants and Study Design

Children and adolescents aged 5 to 15 years from the King Chulalongkorn Memorial Hospital and local communities were enrolled in this study. Children with BMI-SDS exceeding the +2 standard deviation (SD) for age and sex, according to the World Health Organization (WHO) 2007 reference [22], were recruited in the obese group. The recruitment was achieved through the direct approach in the outpatient clinic as well as through social media and the convenient sampling method was adopted. All children were subjected to taking medical history and systemic examination. Children with obesity who had disease or treatment that might affect iron and inflammatory status were excluded from this study, i.e., genetic syndromes (such as Down syndrome, Prader-Willi syndrome), endocrine disorders (such as hypothyroidism, growth hormone deficiency, and Cushing's disease), autoimmune disease, cancer, or receiving iron therapy within three months were excluded. Written informed consent and assents were obtained from the parents and children, respectively, before the study. Stored serum samples of heathy nonobese children who were age- and sex-matched were used as control group. These samples were from the Division of Infectious Diseases, Department of Pediatrics, Faculty of Medicine, Chulalongkorn University. These children and parents have given consent for their stored sample to be used in other research studies.

This cross-sectional study was approved by the Institutional Review Board of the Faculty of Medicine, Chulalongkorn University (IRB no. 352/57).

### 2.2. Methods

Trained personnel performed anthropometric measurements. Body weight and height of each participant were measured while the participant was wearing light clothing and not wearing shoes. Weight and height were measured by using TANITA WB-380H (TANITA. Co., Ltd., Itabashi-ku, Tokyo, Japan), in all participants to the nearest 0.1 kg and 0.1 cm, respectively. This model can measure range of weight and height between 0 and 300 kg and 61–250 cm, respectively. Two measurements were taken and if a difference of weight over 1 kg or height over 1 cm between the measurements occurred, a third measurement was taken and averaged. BMI was calculated as weight in kilograms divided by the square of height in meters (kg/m^2^). BMI-SDS was calculated based on the WHO 2007 growth reference using the WHO AnthroPlus program [23].

Dietary intake was assessed by a dietitian during an interview from a 24-hour dietary recall. Total dietary iron intake was calculated from the amount of iron (mg) per 100 gm of food portion based on the Table of Nutritional Value of Thai Food, published by the Nutrition Division, Ministry of Public Health, Thailand (2001).

Laboratory assessments were performed for all participants. Nonfasting blood samples were obtained for complete blood count (CBC) to measure hemoglobin (Hb), hematocrit (Hct), and mean corpuscular volume (MCV) by using the Coulter LH 780 (Beckman Instrument Incorporation, FL). The serum samples were stored at −20°C for further analysis. The serum iron, ferritin, and total iron binding capacity (TIBC) were assayed using the Cobas 6000 analyzer series (Roche, USA). The percentage of transferrin saturation (TS) was calculated as (serum iron/TIBC) × 100 [24]. Serum hepcidin was assayed using a hepcidin ELISA Kit (Human Hepc25), a sandwich-ELISA Technique provided by Opsys MR Microplate Reader (DYNEX Technologies, USA). The Curve expert 1.4 software was used for calculation. Detection range of serum hepcidin was between 1.56 and 100 ng/mL.

ID was defined when TS was 15% and below [24]. Anemia was defined when Hb was below the age- and sex-matched reference [25]. Children who met the criteria for ID but their Hb was above the reference value (age- and sex-matched) were considered to have ID without anemia.

This study calculated the sample size based on prior report [20]. We assumed that a mean hepcidin-25 is 31.76 ng/mL with a standard deviation of 8.4 ng/mL, using a 2-tailed *t*-test with a type I error probability of 0.05. A sample size of 30 cases and a minimum of 30 controls would have 80% power to detect a small difference of 6 ng/mL between cases and controls.

### 2.3. Statistical Analysis

The normality of data was evaluated using the Shapiro-Wilk test. Data were expressed as frequencies and percentage for categorical variables. Median and interquartile ranges (IQR) were used for continuous variables. The Wilcoxon rank-sum test was used to examine the differences in median values between the obese and nonobese groups. Spearman's correlation coefficients were used to evaluate the relationships between iron profiles and BMI-SDS and between iron profiles and serum hepcidin-25. The Kruskal–Wallis test was preformed to assess the differences between the median values among the children with obesity and ID, obesity without ID, and controls. All statistical tests were two-sided and statistical significance was defined as *p* value < 0.05. Data analyses were performed using Stata version 13.1 (Stata Corp., College Station, Texas).

## 3. Results

### 3.1. Anthropometrical and Biochemical Features

There were 63 children and adolescents with obesity in the obese group [median (IQR) age 10 (9–13) years, 66.7% male] and 27 healthy nonobese children and adolescents in the control group [median (IQR) age 9 (7–12) years, 59.3% male]. Age, gender, anthropometry, and iron profiles were compared between the groups (Table 1). The median BMI-SDS of the obese group was 2.3 (2.0–2.6) versus -0.5 ((-1.3)-0.4) for the nonobese group. There were no significant differences in age and gender between the two groups. Of the 63 children in the obese group, 27 children (42.9%) were ID and 4 children (6.3%) were ID with anemia. However, 2 children with obesity (3.2%) had anemia without ID. Hb, Hct, and MCV showed no differences between the groups. Serum iron and ferritin were slightly higher, and TIBC was much higher in the obese group compared with the control group. TS of the obese group was much lower than the control group. Serum hepcidin-25 of the obese group was significantly higher than the control group.

### 3.2. Associations of BMI-SDS with Iron Profiles and Serum Hepcidin-25 in All Children

**Figure 1:**
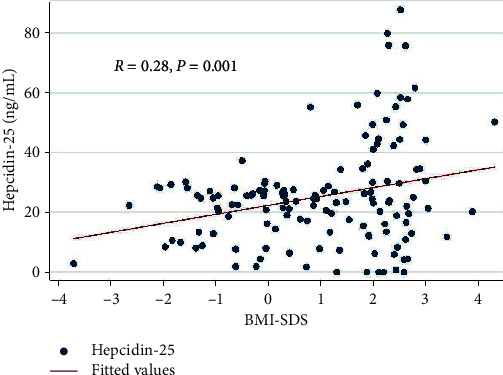
Correlation between serum hepcidin-25 and BMI-SDS.

A positive correlation between BMI-SDS and hepcidin-25 (*r* = 0.28, *p* = 0.001) is shown in Figure 1. The BMI-SDS showed a strongly significant positive correlation with TIBC and a negative association with TS. In addition, only hepcidin-25 was positively correlated with TIBC (Table 2). Age was not associated with an increased risk of ID in the children with obesity (data not shown).

### 3.3. Clinical and Laboratory Data of Children with Obesity and ID, Children with Obesity without ID, and Controls

In the obese group, there was no significant difference in BMI-SDS between the children with ID and without ID (Table 3). There were no differences in Hb and Hct between the subgroups of the girls who already had menstrual cycles and those who did not (data not shown). Ferritin was significantly higher in the obese group than controls and the highest ferritin was found in the children with obesity without ID. The children with obesity without ID had significantly higher serum iron than the nonobese controls. TIBC of the children with obesity, both with and without ID, were significantly higher than controls. The median value of iron intake in children with obesity was 6.6 (5.5–7.8) mg/day.

**Figure 2:**
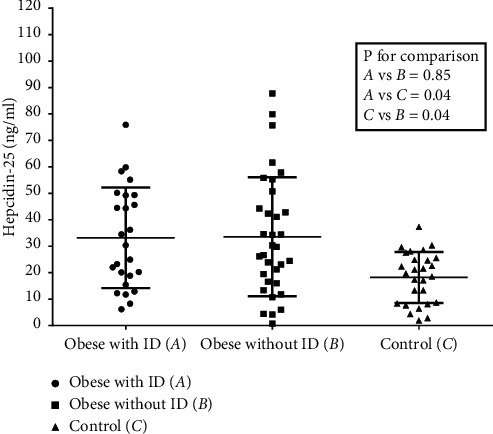
Comparisons of serum hepcidin-25 between three groups.

Serum hepcidin-25 levels of the children with obesity, both with and without ID, were significantly higher than in controls (Figure 2). There were no significant differences in hepcidin-25 and iron intake of the children with obesity between those with ID and those without ID.

## 4. Discussion

The prevalence of ID in children with obesity in this study was 42.9%. This prevalence was consistent with previous studies [2,8] but higher than the prevalence of ID in 6- to 12-year-old Thai children (32.4%) from the SEANUTS study [26]. The proportion of ID with anemia in the obese group in this study was 6.3% which was also higher than in normal school-aged Thai children (5.1% from the SEANUTS study). Incidentally, there were two children in our study who had anemia without ID, which could be due to hemoglobinopathy. Our study also revealed that the dietary iron intake [median 6.6 (5.5–7.8) mg/day] in the children and adolescents with obesity was lower than the Thai Dietary Reference Intake of 11.8 mg/day [27].

High serum ferritin was found in the children and adolescents with obesity in our study. This result was concordant with that of Yimyaem et al. [8] from the north-eastern part of Thailand. In general, ferritin level varies according to age. The level is high at birth, rises during the first two months of life, and then falls consistently in the later infancy stage [19]. At about one year of age, the concentration begins to rise again and continues to increase into adulthood [28]. Ferritin is an acute-phase protein that is upregulated during infections, inflammatory states, and malignant diseases. It is suggested that serum ferritin level is elevated in response to inflammation in obesity, even in the persons with ID [29]. Therefore, we proposed that ferritin level alone was not a good indicator for iron status in children with obesity and defined the ID status by using TS, the quotient of serum iron, and TIBC. TS equal to 15% and below reflected an inadequate iron intake based on the normal daily requirement, and a prolonged period of ID resulted in an inadequate erythropoiesis which led to changes in the number and shape of newly released reticulocytes and erythrocytes [24].

Our study revealed higher serum hepcidin-25 in children and adolescents with obesity compared with the normal weight controls. This finding is similar to a recent study by Sal et al. [21]. In addition, study from Sanad et al. [30] revealed that serum hepcidin-25 was positively correlated with TIBC but negatively correlated with Hb, serum iron, and TS. However, we could only demonstrate the positive correlation between hepcidin-25 and TIBC in our children. Although previous studies revealed negative correlations between the BMI-SDS and many iron profile abnormalities [20, 31–33], our study found that BMI-SDS correlated positively with TIBC and negatively with TS. According to this finding, it proved higher body weight to be lower iron status. Furthermore, there was a weak correlation between the BMI-SDS and hepcidin-25 (*r* = 0.28, *p* = 0.001) from all subjects which included both the children with obesity and controls, which was supported by previous studies [21, 31]. In subgroup of children with obesity, there was no significant difference in hepcidin-25 between obese with ID and without ID. This finding was consistent with the most recent study by Nashar et al. [34], where the researchers also reported that hepcidin-25 in obese children with ID did not differ from those without ID. This result might be explained by Chang et al. [35] who showed that children with overweight and obesity had lower interleukin-10 (IL-10) compared to children with normal weight. Low serum IL-10 leads to increased IL-6 production that is the most potent hepcidin regulator. It is possible that imbalance of inflammatory cytokine results in elevated serum hepcidin in both obese children with ID and without ID. Therefore, our study confirmed that children and adolescents with obesity or those with higher BMI-SDS have higher hepcidin. Nevertheless, we failed to demonstrate the association between the increased hepcidin and ID in this population.

There are several explanations regarding the cause of ID in children with obesity, for example, inadequate iron intake, higher iron requirement due to higher body mass or blood volume, and lower myoglobin in the muscle due to a lack of physical activity [36]. Children with obesity had a large scale of adipocytes, which promoted macrophage to accumulate and release of the proinflammatory cytokines, including IL-6, IL-1*β*, and TNF-*α*, which showed some degree of chronic inflammation [9–12]. The releases of these cytokines mainly result in the release of hepcidin from the liver or adipose tissues [13]. Hepcidin-25 is an important regulator of iron homeostasis. It inhibits intestinal iron absorption and inhibits iron release from macrophages which leads to decreased iron status, hypoferremia, and anemia of chronic disease [15, 16, 37, 38]. These findings suggest that inflammation may perpetuate hepcidin-mediated inhibition of dietary iron absorption, leading to ID [39]. Therefore, lower bioavailability of iron among adults with obesity may potentially be related to greater adipose tissue expression of hepcidin [40]. Although hepcidin expression is more than 100-fold higher in hepatocytes than in adipocytes, hepcidin secreted from both tissues may be relevant because the adipose tissue mass in obesity may be 20 times greater than liver mass [41]. In addition, study from Amato et al. [32] revealed that significant decrease in serum hepcidin after weight loss program can improve iron status and iron absorption in children with obesity. This study suggested that weight reduction in children with obesity may be an ideal management for correction of ID. From this pathophysiology, the high serum hepcidin-25 and poor dietary iron intake may be contributing factors, not just mediators, to ID in our Thai children and adolescents with obesity. Regrettably, due to our small sample size, we cannot firmly prove that hepcidin-25 contributed to ID.

Other methodological limitations of the current study included random blood sampling which may have interfered with serum hepcidin due to diurnal variation [42]. Secondly, we did not measure other inflammatory markers associated with hepcidin-25 or degree of obesity, such as CRP, IL-6, or erythrocyte sedimentation rate. Moreover, this was an observational study; thus it could not prove the causation between the demonstrated associations. We suggest further investigation in a larger sample size and/or with interventions either to reduce the degree of obesity or to improve iron intake and evaluate whether the interventions could correct the ID.

## 5. Conclusions

Thai children and adolescents with obesity had a higher prevalence of ID and higher serum hepcidin-25 than children of normal weight. Inadequate dietary iron intake together with chronic inflammation stemming from excess adipose tissue may contribute to the depleted iron status. Further study in a larger population, preferably with interventions such as weight loss program, is warranted to modify or correct a poor iron status in children with obesity.

## Figures and Tables

**Table 1:** Anthropometrical and biochemical features of the obese group versus controls.

Variables	Cases (*N* = 63)	Controls (*N* = 27)	*p* value
Age (years)	10 (9–13)	9 (7–12)	0.41
Sex, male (*n*, %)	42 (66.7)	16 (59.3)	0.67
BMI (kg/m^2^)	28.2 (26.1–33.2)	15.7 (14.1–18.0)	<0.001
BMI-SDS	2.3 (2.0–2.6)	−0.5 ((−1.3)–0.4)	<0.001
Hb (g/dl)	13.3 (12.8–13.9)	13.2 (12.3–13.9)	0.38
Hct (%)	39.9 (38.2–41.3)	39.2 (37–40.9)	0.39
MCV (fl)	76.6 (74.0–80.0)	76.8 (69.9–83.1)	0.76
Serum iron (mcg/dl)	69.0 (50.0–91.0)	54.0 (38.0–73.0)	0.03
Ferritin (ng/ml)	77.0 (51.4–114.0)	55.0 (35.0–77.1)	0.03
TIBC (mg/dl)	410.0 (377.0–447.0)	193.0 (176.0–323.0)	<0.001
Transferrin saturation (%)	15.2 (12.5–25.6)	26.6 (17.0–29.4)	0.001
Hepcidin-25 (ng/ml)	26.2 (12.9–45.6)	19.7 (8.3–25.5)	0.02

BMI: body mass index; BMI-SDS: body mass index-standard deviation score; Hb: hemoglobin; Hct: hematocrit; MCV: mean corpuscular volume; TIBC: total iron binding capacity. All data were expressed as median (IQR) except sex (*n*, %); *p* value from Wilcoxon rank-sum test.

**Table 2:** Correlations between BMI-SDS, serum hepcidin-25, and iron profiles in all children.

Variables	Hb (95% CI)	Hct (95% CI)	Serum iron (95% CI)	Ferritin (95% CI)	TIBC (95% CI)	TS (95% CI)
BMI-SDS	0.02 ((−0.18)−0.21)	−0.01 ((−0.22)−0.18)	0.20 ((−0.01)−0.38)	0.15 ((−0.08)−0.35)	0.52^*∗∗*^ (0.31–0.68)	−0.28^*∗*^ ((−0.47)−(−0.09))
Hepcidin-25	−0.01 ((−0.24)−0.21)	0.05 ((−0.20)−0.27)	0.16 ((−0.07)−0.38)	0.17 ((−0.04)−0.36)	0.34^*∗*^ (0.16–0.49)	−0.12 ((−0.33)−0.09)

Data represent the correlation coefficient (*r*) and 95% confidence interval (CI), ^*∗*^*p* value < 0.05; ^*∗∗*^*p* value < 0.001 from Spearman's correlation test. Hb: hemoglobin; Hct: hematocrit; TIBC: total iron binding capacity; TS: transferrin saturation.

**Table 3:** Comparison of BMI-SDS, iron profiles, and serum hepcidin-25 among children with obesity and ID, children with obesity without ID, and controls.

	Obese with ID (*A*) (*N* = 27)	Obese without ID (B) (*N* = 36)	Controls (C) (*N* = 27)	*p* value^†^ overall	*p* value^§^ for A vs. B	*p* value^§^ for A vs. C	*p* value^§^ for B vs. C
BMI-SDS	2.3 (2.0–2.7)	2.4 (2.0–2.6)	−0.5 ((−1.3)−0.4)	<0.001	0.81	<0.001	<0.001
Hb (g/dl)	13.1 (12.5–13.9)	13.4 (12.8–14.1)	13.2 (12.3–13.9)	0.43	0.35	0.84	0.22
Hct (%)	39.2 (37.9–41.1)	39.9 (38.4–41.4)	39.2 (37–40.9)	0.57	0.53	0.72	0.29
Serum iron (mcg/dl)	48 (42–57)	87.5 (70–106.5)	54 (38–73)	<0.001	<0.001	0.30	<0.001
Ferritin (ng/ml)	77 (50.4–114)	82.2 (52.3–107.9)	55 (35–77.1)	0.08	0.96	0.06	0.04
TIBC (mg/dl)	414 (388–487)	403 (349.5–438)	193 (176–323)	<0.001	0.10	<0.001	<0.001
Transferrin saturation (%)	12.1 (10.5–14.6)	23.4 (17.7–27.1)	26.6 (17–29.4)	<0.001	<0.001	<0.001	0.55
Hepcidin-25 (ng/ml)	25 (12.9–49.2)	26.4 (12.6–43.6)	19.7 (8.3–25.5)	0.06	0.85	0.04	0.04
Iron intake (mg/day)	6.6 (5.1–7.8)	6.7 (5.8–7.7)	NA	0.73	NA	NA	NA

All data were expressed as median (IQR), BMI-SDS: body mass index-standard deviation score; Hb: hemoglobin; Hct: hematocrit; MCV: mean corpuscular volume; TIBC: total iron binding capacity; ^†^*p* value for compared median between the obese with ID, obese without ID, and control using Kruskal–Wallis test. ^§^*p* value for compared median between two groups using Wilcoxon rank-sum test.

## Data Availability

The data used to supporting the findings of this study are available from the corresponding author upon request.
